# Corrosion Resistance and Mechanism of Four 6Mg Zinc-Based Coatings: A Comparative Study of 15Al, 17Al, 19Al, and 22Al

**DOI:** 10.3390/ma19142976

**Published:** 2026-07-10

**Authors:** Yuanpeng Li, Zhao Li, Sheming Jiang, Jie Zhang, Qifu Zhang

**Affiliations:** 1 National Engineering Laboratory of Advanced Coating Technology for Metals, Central Iron & Steel Research Institute, Beijing 100081, China; 15332811094@163.com (Z.L.);; 2 Beijing Baogang Steel Technology Co., Ltd., Beijing 100083, China

**Keywords:** high-magnesium Zn-Al-Mg, corrosion resistance, corrosion mechanism, microstructure

## Abstract

**Highlights:**

- The planar corrosion resistance, edge corrosion resistance, and punched-hole corrosion resistance of Zn–6%Mg-based coatings with varying Al contents were compared.
- The corrosion products of high-magnesium Zn–Al–Mg coatings after different cyclic corrosion test periods were analyzed.
- The cross-sectional microstructures of high-magnesium Zn–Al–Mg coatings after different cyclic corrosion test periods were examined, and the corresponding corrosion resistance mechanism was elucidated.

**Abstract:**

High-magnesium Zn-Al-Mg coatings with different Al contents were prepared using a laboratory hot-dipped galvanizing simulator. The planar corrosion resistance and its mechanism were systematically investigated by analyzing the microstructure and corrosion products at different corrosion cycles. Cyclic corrosion test results show that the planar corrosion resistance does not increase monotonically with higher Al content; the 19Al6Mg coating exhibits the best planar corrosion resistance. Among the four alloy coatings, the 15Al6Mg coating demonstrates the best cut-edge corrosion resistance in both cut-edge and punched Hole corrosion tests. Furthermore, the corrosion mechanism of coatings with different Al contents was analyzed. The types of corrosion products formed on these Zn-Al-Mg coatings are essentially identical. However, as the Al content increases, the proportion of the Al phase and the resulting Al-Zn eutectoid structure increases. During corrosion, the Al-Zn eutectoid structure, which has a lower corrosion potential, corrodes preferentially. This explains why increasing the Al content to 22% leads to a reduction in the overall corrosion resistance of the coating.

## 1. Introduction

Research on steel anti-corrosion technology holds significant scientific and engineering importance for resource conservation and economic benefits. Among various protection technologies, hot-dip galvanizing is the most cost-effective method for steel protection. The zinc coating provides long-term protection for the steel substrate through a physical barrier, sacrificial anodic electrochemical protection [1,2], and the inhibitory effect of corrosion products [3,4]. To further enhance the corrosion resistance of zinc coatings and reduce coating thickness, researchers have continuously explored the addition of alloying elements such as Al and Mg to zinc baths [5].

Since the late 1970s, Japanese scholars discovered that adding Mg to Zn-Al alloys yields Zn-Al-Mg coatings with superior performance, subsequently launching commercial products such as ZAM^®^ [6,7] and SuperDyma^®^ [8]. These coatings feature Al content of 5~11% and Mg content of 3%, belonging to the medium-aluminum system Zn-Al-Mg coatings, with corrosion resistance improved by 4 to 20 times [9,10], and are widely used in construction, home appliances, and other fields [11,12,13]. Although these Zn-Al-Mg alloy coatings exhibit excellent corrosion resistance in high-salt environments [11,14,15,16,17], the increasing market demand for corrosion performance means these coatings can no longer meet the more stringent service conditions of C5, CX [18], and other corrosive environments. Therefore, developing a new generation of high-magnesium Zn-Al-Mg alloy coatings with superior corrosion resistance has become an urgent need in current research.

Recent progress has been made in the development of high-magnesium Zn-Al-Mg alloy coatings. For example, Nippon Steel announced the launch of ZEXEED^®^ coating in 2022 [19], and South Korea subsequently reported the development of PosMac Super^®^ coating [20]. Studies have shown that high-magnesium Zn-Al-Mg coatings exhibit further improved corrosion resistance [19,21,22,23] and show broad industrial application prospects. However, systematic research on the intrinsic correlation between composition design and corrosion resistance remains limited. Therefore, this paper aims to systematically investigate the corrosion resistance and mechanisms of four 6Mg zinc-based coatings (15Al, 17Al, 19Al, and 22Al), providing a theoretical basis and technical support for the industrial production of high-corrosion-resistance Zn-Al-Mg coated steel sheets in China.

## 2. Experimental Method

### 2.1. Materials

**Figure 1:**
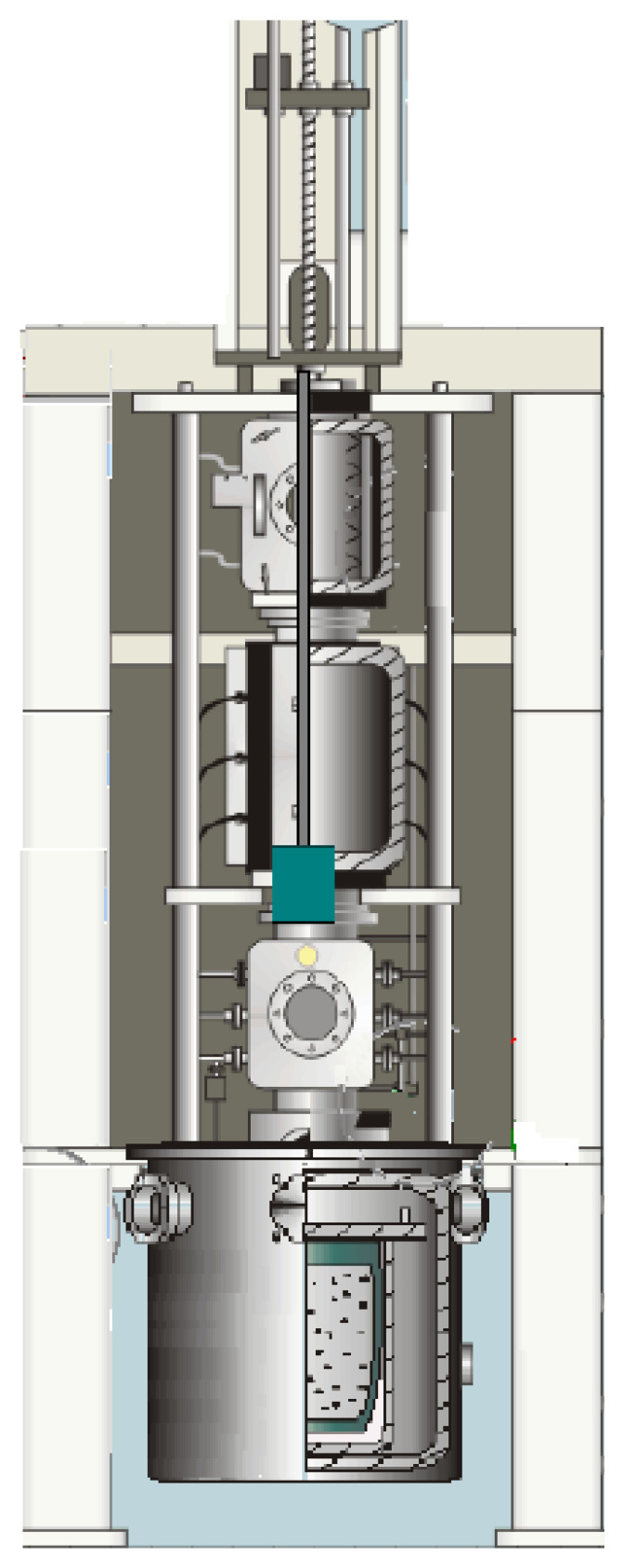
Equipment schematic of the hot-dip galvanizing simulator.

Zn-Al-Mg alloy coating samples were prepared using a laboratory hot-dipped galvanizing simulator. Figure 1 shows the schematic diagram of the hot-dipped galvanizing simulator equipment.

The substrate steel plates, supplied by a Chinese steel mill for its 2030 mm cold rolling line, are commercial low-carbon steel SPCC of 0.8 mm and 2 mm thickness. The 0.8 mm plates are used for planar corrosion tests, and the 2 mm plates for cut-edge corrosion tests. Table 1 presents the detailed chemical composition of the SPCC substrate steel plate. The specific composition of the steel plate is provided by the steel mill. During the experiment, the specimen dimensions are 110 mm × 220 mm, with the dipping direction consistent with the rolling direction of the steel sheet.

In this study, Pandat 2024 software was used to calculate the melting points of the corresponding alloys, and the bath temperatures were set based on these melting points. Table 2 shows the melting points of different alloy components and the bath temperatures.

To obtain alloy-coated steel sheet samples with good surface quality and adhesion, the substrate steel sheet underwent multi-stage cleaning treatment before laboratory hot-dip galvanizing tests to remove surface iron powder, residual oil, and oxide film [24]. The process included: soaking in boiling deionized alkaline solution at 100 °C for more than 30 min, followed by alkaline solution brushing, hot water brushing, and two deionized water rinses. The rinsed substrate steel sheet was wiped with clean tissue paper, dried with a blow dryer, and finally stored in a desiccator to ensure the surface cleanliness required for hot-dip galvanizing.

Since the substrate steel sheet material was identical, the same annealing process was adopted during laboratory hot-dip galvanizing. The temperature was ramped up at 10 °C/s to 750 °C under 90% N_2_ and 10% H_2_ atmosphere, soaked at 750 °C for 50 s, then cooled at 30 °C/s to the specified temperature under 70% N_2_ and 30% H_2_ atmosphere, dipped into the zinc bath for 3 s to complete galvanizing. After leaving the bath, the coated steel sheet was cooled at 10 °C/s to 200 °C under 70% N_2_ and 30% H_2_ atmosphere, then removed and air-cooled to room temperature. During the experiment, high-purity nitrogen was used to displace the air in the simulator, ensuring the oxygen concentration during hot-dip galvanizing was strictly controlled below 20 ppm. To precisely monitor temperature changes during dipping, a thermocouple was welded on the back of the substrate, 35 mm from the top edge. The direction of immersion of the steel sheet into the zinc bath was consistent with the rolling direction.

Differences in bath composition directly affect hot-dip galvanizing process parameters, including the steel sheet entry temperature and bath temperature. Table 3 summarizes the hot-dip galvanizing process parameters corresponding to alloy-coated steel sheets with different Al compositions. Given that alloys with different Al contents have different melting points, to ensure good bath fluidity, the bath temperature is typically controlled at 40~50 °C above the alloy melting point in production. Meanwhile, to avoid local solidification of the zinc bath due to excessive temperature difference upon entry, the steel sheet temperature before immersion should be 20 °C higher than the bath temperature.

### 2.2. Characterization

#### 2.2.1. Cyclic Corrosion Test

**Figure 2:**
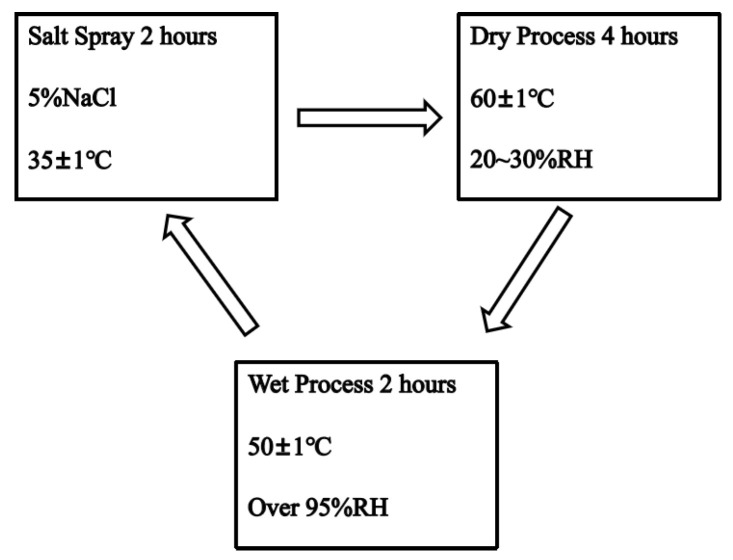
Detail of the CCT cycle.

Before cyclic corrosion testing, the coated steel sheets were cut into rectangular specimens of 70 mm × 70 mm × 0.8 mm using a wire cutting machine, ensuring at least three parallel specimens per coating type. The specimens were cleaned with anhydrous ethanol, dried with a blow dryer, and then placed in a salt spray corrosion test chamber. Cyclic corrosion tests were conducted according to the JASO M 609-91 standard [25], continuing for more than 300 cycles until red rust appeared on the specimen surface. The corrosive medium used was 5 wt% NaCl aqueous solution. Details of the cyclic test sequence are shown in Figure 2.

#### 2.2.2. X-Ray Diffraction Analysis

Phase analysis of corrosion products on coating specimens was performed using a German Bruker D8 ADVANCE X-ray diffractometer (Bruker AXS GmbH, Karlsruhe, Germany). The XRD patterns contain information such as diffraction peak positions, shapes, and intensities. MDI Jade 9 software was used to compare with ICDD standard PDF 2009 cards for phase identification of characteristic diffraction peaks. For corrosion product analysis, either the original specimens (for shorter corrosion times) or specimens with spalled corrosion products (for longer corrosion times) were used. The target was Cu, with a tube voltage of 40 kV, tube current of 40 mA, and scanning speed of 6°/min. To avoid interference from air peaks, the scanning range for corrosion product analysis was 2θ = 5°~90°.

#### 2.2.3. SEM/EDS Microstructural Characterization and Composition Analysis

A FEI QUANTA FEG 650 field emission scanning electron microscope (FEI, Hillsboro, OR, USA) equipped with a backscattered electron detector and X-ray energy dispersive spectrometer was used to analyze the cross-sectional microstructure of alloy-coated specimens after different corrosion cycles. The alloy-coated steel sheets after different corrosion cycles were cut into small specimens of 10 mm × 10 mm and cold-mounted using acrylic powder. The specimen surfaces were ground sequentially with 120#, 240#, 320#, 400#, 600#, 800#, 1000#, and 1500# SiC waterproof abrasive paper. Each time a finer grit paper was used, the specimen was rotated 90° to eliminate deep scratches from the previous step until the surface showed a uniform frosted appearance. After grinding, the specimens were polished using anhydrous ethanol as coolant and diamond suspension polishing fluid with a particle size of 2.5 μm on a polishing machine until the surface was smooth, scratch-free, and exhibited a mirror finish. Subsequently, backscattered electron imaging mode was used at an operating voltage of 20 kV and working distance of 10 mm to observe the cross-sectional microstructure of alloy-coated specimens after different corrosion cycles. Meanwhile, the X-ray energy-dispersive spectrometer attached to the SEM was used for qualitative and semi-quantitative analysis of elemental composition in selected areas.

## 3. Results

### 3.1. Planar Corrosion Resistance

**Figure 3:**
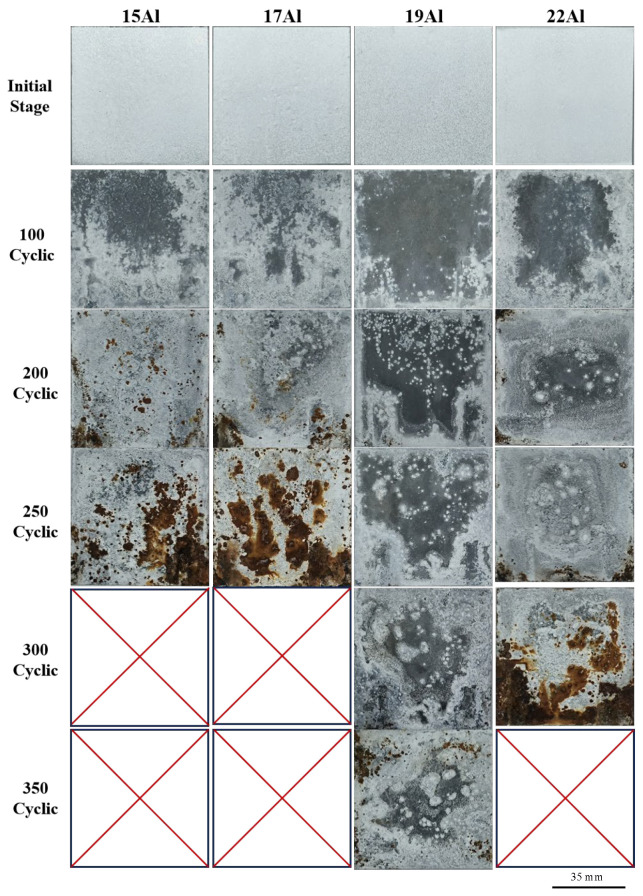
Planar corrosion results of cyclic salt spray test for four coatings under different Al contents.

In this study, a wet-dry alternating cyclic corrosion test method that more closely simulates the natural corrosion environment was adopted, following standard JASO M 609-91. Each test cycle consisted of 2 h of salt spray, 4 h of drying, and 2 h of wetting (see Figure 2). Figure 3 shows the cyclic corrosion test results for the four coatings with different Al contents. The results show that the overall corrosion resistance of the 15Al6Mg coating and the 17Al6Mg coating is similar, with both showing red rust after 250 cycles. The 19Al6Mg coating began to show red rust after 350 cycles. The 22Al6Mg coating showed obvious red rust after 300 cycles. When the Al content does not exceed 19%, the corrosion resistance of the Zn-Al-Mg alloy coating increases with increasing Al content. However, higher Al content does not necessarily mean better coating corrosion resistance. When the Al content in the coating reaches 22%, the coating corrosion resistance decreases.

### 3.2. Cut-Edge Corrosion Resistance

**Figure 4:**
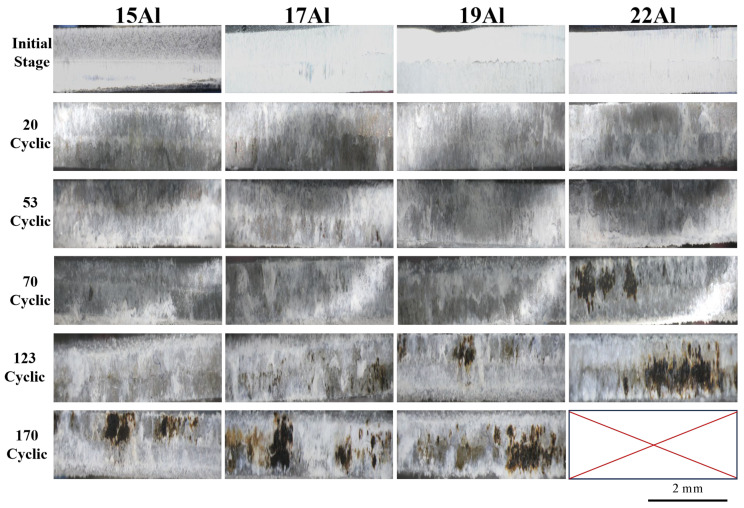
Cut edge corrosion results of cyclic salt spray test for four coatings under different Al contents.

Figure 4 shows the cyclic salt spray cut-edge corrosion test results for the four coatings with different Al contents under a cut thickness of 2 mm. The data show that the 15Al6Mg coating only showed red rust after 170 cycles, exhibiting the best cut-edge corrosion resistance, while the 22Al6Mg coating began to show red rust after only 70 cycles, demonstrating the worst cut-edge corrosion resistance. The 17Al6Mg and 19Al6Mg coatings both began to show red rust after 120 cycles, with the red rust area further increasing after 170 cycles. Among these, the cut-edge corrosion resistance of 17Al6Mg is better than that of 19Al6Mg. These results indicate that in Zn-Al-Mg coatings, Al content has a detrimental effect on cut-edge corrosion resistance.

### 3.3. Punched Hole Corrosion Test

**Figure 5:**
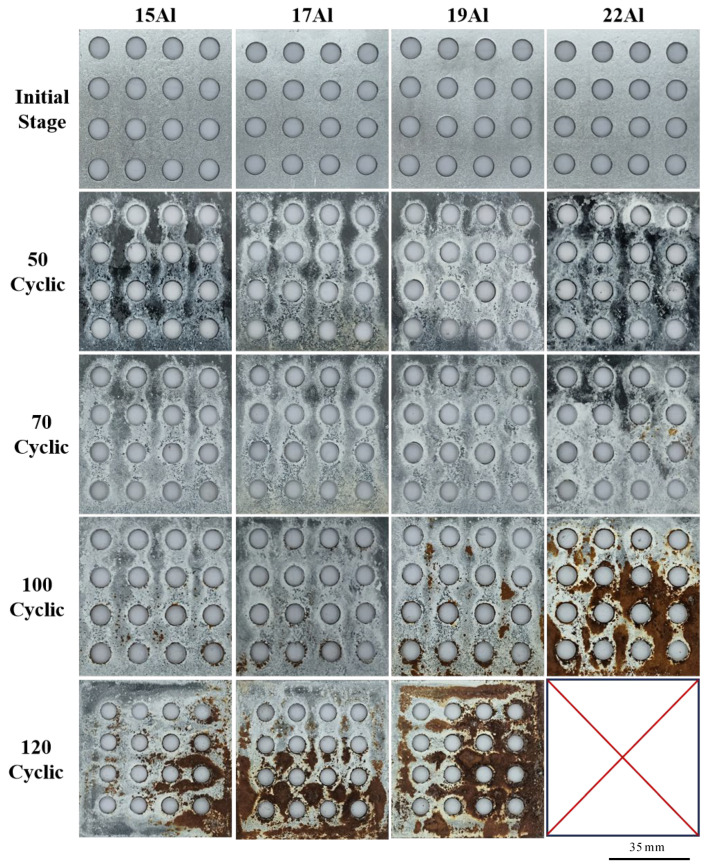
Cyclic salt spray punched hole corrosion test results of coatings with different Al contents.

Figure 5 shows the results of cyclic salt spray punched Hole corrosion tests for the four coatings with different Al contents. The data show that the 22Al6Mg coating exhibits the worst punched Hole corrosion resistance, showing obvious red rust after 100 cycles. In contrast, the 15Al6Mg, 17Al6Mg, and 19Al6Mg coatings only showed significant red rust after 120 cycles. Comparing the amount of red rust generated between 100 and 120 cycles, it can be seen that among the three coatings (15Al6Mg, 17Al6Mg, and 19Al6Mg), 15Al6Mg has the best punched Hole corrosion resistance. These results indicate that in the Zn-Al-Mg coating system, as Al content increases, the punched Hole corrosion resistance of the coating tends to decrease. The reason for the above phenomenon is related to the cut-edge protection of the alloy coating. Specifically, Al content has a detrimental effect on the cut-edge corrosion resistance of the coating. Therefore, with the same cut exposure area, coatings with higher Al content exhibit poorer punched Hole corrosion resistance.

### 3.4. Phase Analysis of Corrosion Products

**Figure 6:**
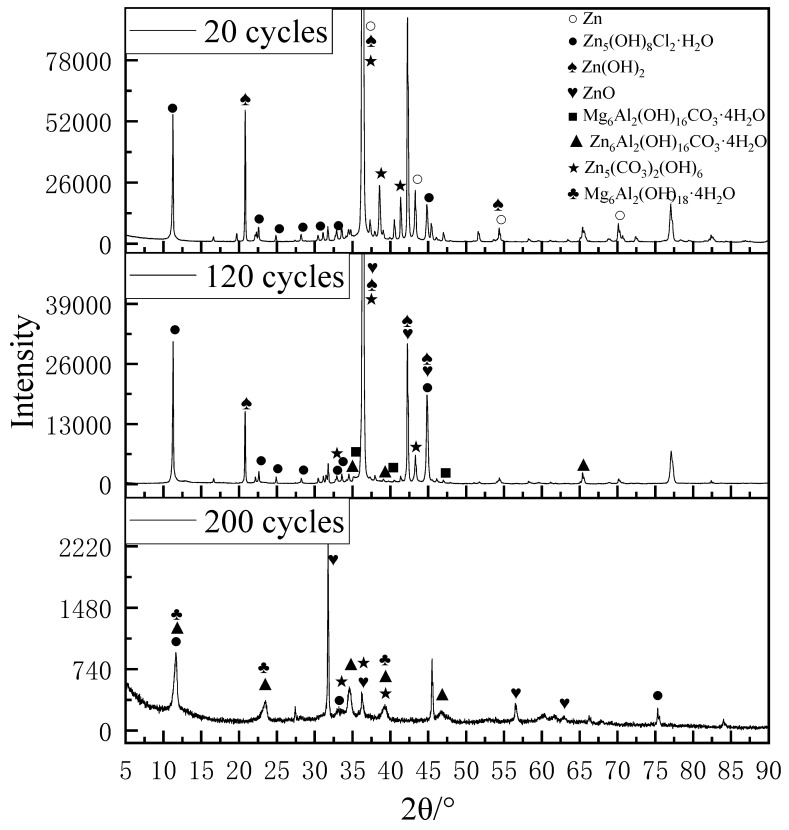
Phase analysis of corrosion products on 15Al6Mg after different cyclic corrosion periods.

Figure 6 shows the phase analysis results of corrosion products on the 15Al6Mg coating after different cyclic corrosion cycles. After 20 cycles, the corrosion products were Zn_5_(OH)_8_Cl_2_·H_2_O, Zn(OH)_2_, and Zn_5_(CO_3_)_2_(OH)_6_. The presence of Zn(OH)_2_ is attributed to the formation of zinc hydroxide in humid air, while ZnO forms on the coating surface in dry air at room temperature. After 120 cycles, the corrosion products were Zn_5_(OH)_8_Cl_2_·H_2_O, Zn(OH)_2_, ZnO, Zn_5_(CO_3_)_2_(OH)_6_, Mg_6_Al_2_ (OH)_16_CO_3_·4H_2_O and Zn_6_Al_2_(OH)_16_CO_3_·4H_2_O. After 200 cycles, the corrosion products were mainly Zn_5_(OH)_8_Cl_2_·H_2_O, ZnO, Zn_5_(CO_3_)_2_(OH)_6_, Zn_6_Al_2_(OH)_16_CO_3_·4H_2_O and Mg_6_Al_2_(OH)_18_·4H_2_O. The corrosion product Mg_6_Al_2_(OH)_18_·4H_2_O is synthesized in a CO_2_-poor environment but is unstable in air and easily adsorbs CO_2_, transforming into Mg_6_Al_2_(OH)_16_CO_3_·4H_2_O.

**Figure 7:**
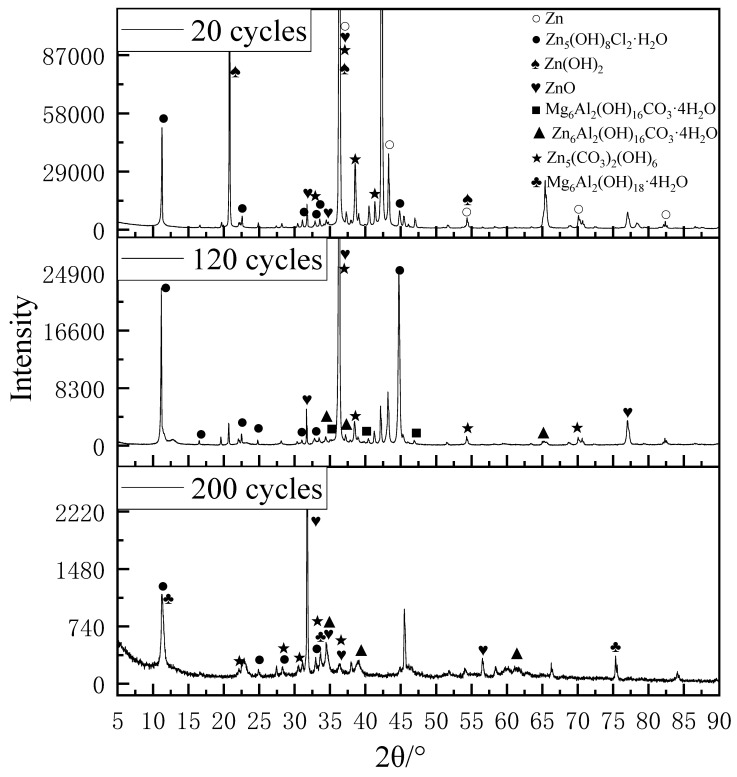
Phase analysis of corrosion products on 17Al6Mg after different cyclic corrosion periods.

Figure 7 shows the phase analysis results of corrosion products on the 17Al6Mg coating after different cyclic corrosion cycles. The corrosion products of the Zn-17%Al-6%Mg coating after 20 and 120 cycles were essentially the same as those of the Zn-15%Al-6%Mg coating. With prolonged corrosion time, Zn_6_Al_2_(OH)_16_CO_3_·4H_2_O and Mg_6_Al_2_(OH)_18_·4H_2_O were detected in the corrosion products. After 200 cycles, the peak of ZnO in the corrosion products gradually increased.

**Figure 8:**
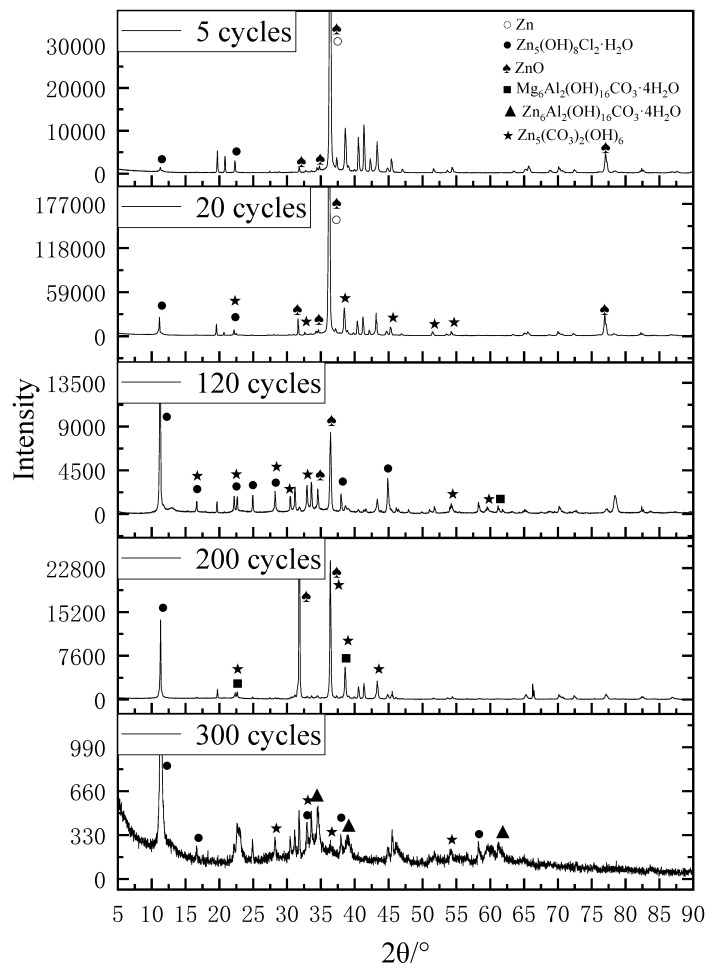
Phase analysis of corrosion products on 19Al6Mg after different cyclic corrosion periods.

Figure 8 shows the XRD diffraction patterns of corrosion products on the 19Al6Mg coating after cyclic corrosion testing. The corrosion products detected after 5 cycles were mainly ZnO and Zn_5_(OH)_8_Cl_2_·H_2_O. The corrosion products after 20 cycles were basically similar to those after 3 cycles, but an obvious Zn_5_(CO_3_)_2_(OH)_6_ peak appeared. After 120 cycles, Mg_6_Al_2_ (OH)_16_CO_3_·4H_2_O was additionally detected in the corrosion products. After 200 cycles, the peak of Mg_6_Al_2_ (OH)_16_CO_3_·4H_2_O increased, while the detected Zn_5_(OH)_8_Cl_2_·H_2_O peak decreased. After 300 cycles, Zn_6_Al_2_(OH)_16_CO_3_·4H_2_O additionally appeared in the corrosion products. This change pattern is basically consistent with the research results of Kohei Tokuda et al. [22] on the corrosion resistance of Zn-19%Al-6%Mg coatings.

**Figure 9:**
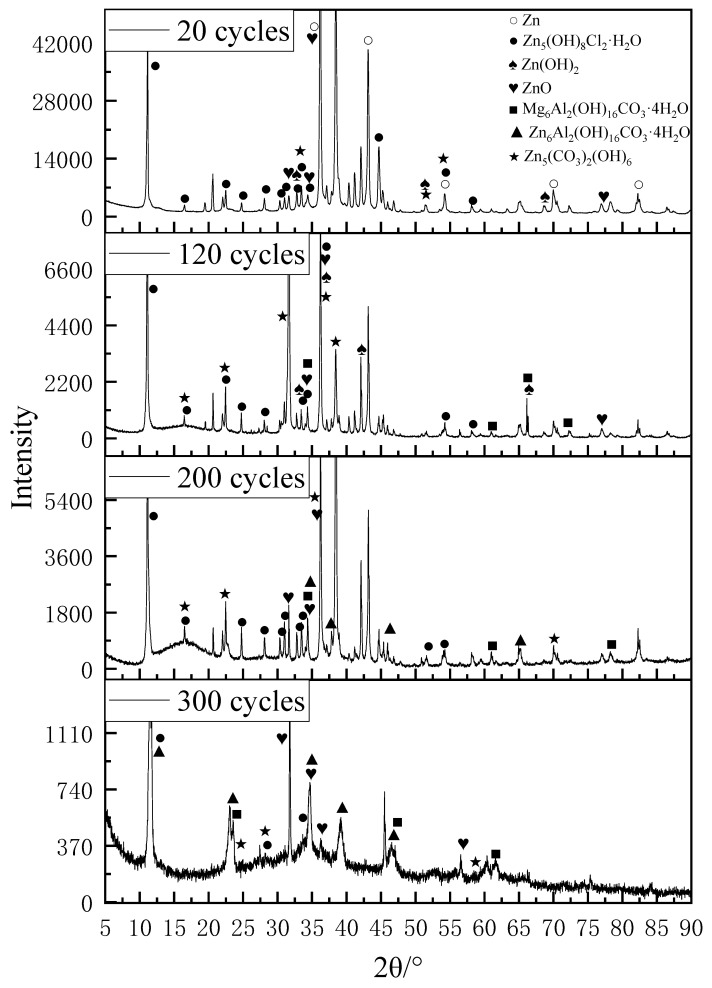
Phase analysis of corrosion products on 22Al6Mg after different cyclic corrosion periods.

Figure 9 shows the phase analysis results of corrosion products on the 22Al6Mg coating after different cyclic corrosion cycles. Unlike the 19Al6Mg coating, Mg_6_Al_2_ (OH)_16_CO_3_·4H_2_O was detected after 120 cycles of cyclic corrosion for the 22Al6Mg coating, and Zn_6_Al_2_(OH)_16_CO_3_·4H_2_O was additionally detected after 200 cycles. Comparing the corrosion products of the four coatings after different cyclic corrosion cycles, it can be found that under cyclic corrosion test conditions, the types of corrosion products formed on high-magnesium Zn-Al-Mg coatings with different Al contents are the same, with only differences in the cycles at which each corrosion product appears.

### 3.5. Corrosion Mechanism

**Figure 10:**
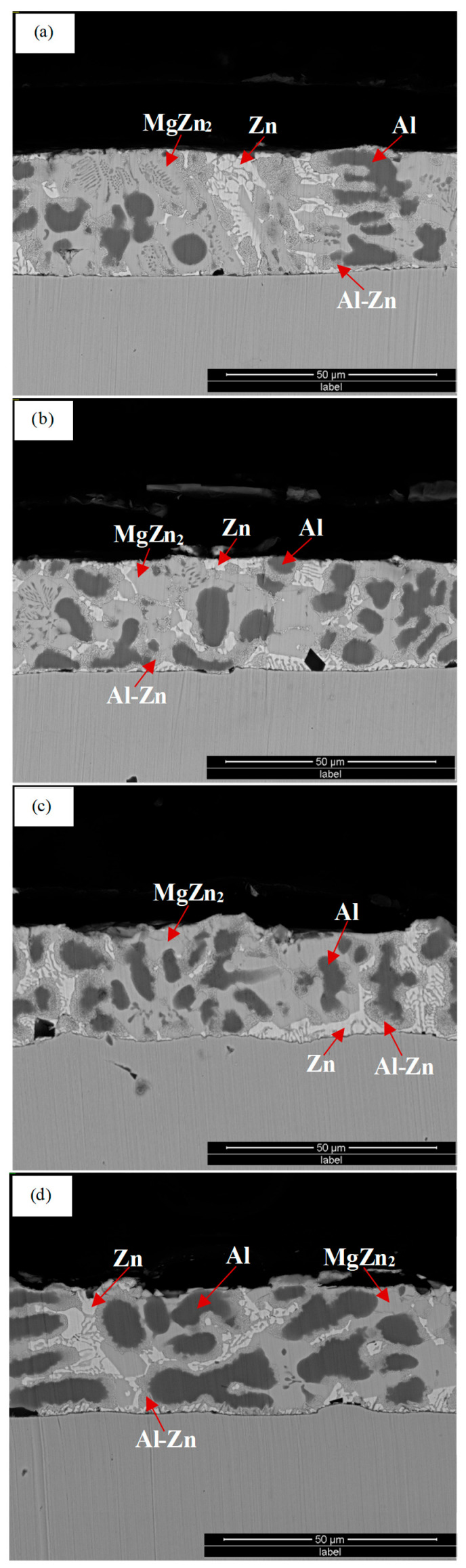
Cross-sectional microstructure of coatings with different Al contents. (**a**) 15Al, (**b**) 17Al, (**c**) 19Al, (**d**) 22Al.

To further elucidate the influence mechanism of the Al element on the corrosion resistance of high-magnesium Zn-Al-Mg alloy coatings, a detailed comparative analysis of the microstructure of Zn-Al-Mg coatings with four different Al contents was conducted. Figure 10 shows backscattered electron images of cross-sections of Zn-Al-Mg coatings with different Al contents at 3000× magnification. The data show that the cross-sectional microstructures of the four coatings all consist of Al phase, Zn phase, and MgZn_2_ phase, with the Al phase surrounded by MgZn_2_ phase [26,27]. Granular or lamellar Al-Zn eutectoid structures exist around the Al phase, and the proportion of primary Al increases with increasing Al content in the coating [28].

As the Al content in the coating increases, the fundamental reason for the reduced corrosion resistance at sheared edges and punched holes is as follows: the proportion of Zn-rich and Mg-rich phases—which provide effective sacrificial anode protection—decreases in the microstructure, while the proportion of Al-rich phases—which tend to passivate in corrosive environments—increases. In common corrosive environments such as neutral, weakly acidic, or weakly alkaline conditions, Zn and Mg remain active and preferentially dissolve, thereby providing effective cathodic protection to the exposed Fe substrate. In contrast, Al forms a dense passive film on its surface, which hinders its own dissolution and its ability to supply electrons outward, thus making it incapable of substituting for Zn and Mg in performing the sacrificial anode protection function.

During the planar corrosion, the Al-rich dendritic phases formed in the coating act as a skeleton, effectively separating Zn- and Mg-rich regions that are prone to corrosion. This significantly hinders the penetration of water and oxygen toward the steel substrate, thereby improving the planar corrosion resistance. Existing studies have generally pointed out that increasing Al content in the coating helps improve its corrosion resistance [9,10,11,12,13,17,29,30,31,32].

However, the relationship between Al content and the corrosion resistance of Zn-Al-Mg coatings is not simply linear [33]. From the cross-sectional structures of the four coatings shown above, it can be clearly observed that the primary Al phase is accompanied by a large amount of Al-Zn eutectoid structure. As the proportion of Al phase increases, the proportion of Al-Zn eutectoid structure also increases.

Table 4 shows the corrosion potential data for Al-Zn alloy coatings in 3 wt% NaCl aqueous solution: under steady-state conditions, the corrosion potential of pure Al is approximately −1.12 V, Al-78%Zn alloy approximately −1.10 V, and pure Zn approximately −1.03 V [19,34,35]. Although the electrochemical potential of Al is more negative than that of Zn (approximately −1.12 V), in neutral or weakly acidic environments, Al easily forms a dense alumina film on its surface, causing its actual potential to rise relatively. It is worth noting that Zn-Al alloys with a composition close to Al-78%Zn exhibit a lower corrosion potential than pure Zn. Other studies have also confirmed that the combination of Al and Zn lowers the overall potential of the alloy. During coating corrosion, corrosion typically begins with the phase having the lower potential. When the Zn-Al eutectoid structure at the edges of primary Al preferentially corrodes due to its lower potential, it will adversely affect the overall corrosion resistance of the coating (Table 4).

**Figure 11:**
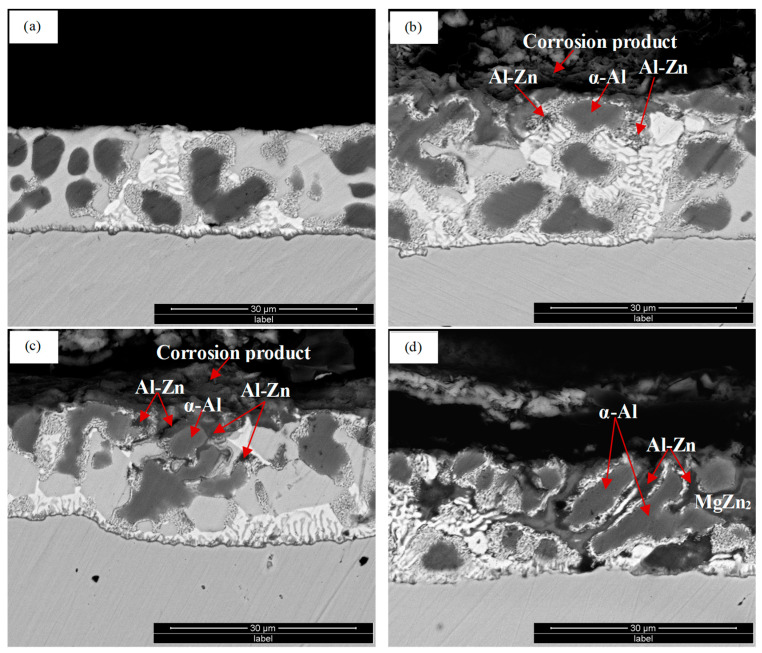
Cross-section microstructure of the 19Al6Mg after different cyclic corrosion periods; (**a**) 5 cycles, (**b**) 100 cycles, (**c**) 120 cycles, (**d**) 150 cycles.

By comparing the cross-sectional microstructure of the 19Al6Mg coating after different cycles of cyclic corrosion testing (as shown in Figure 11), significant differences in the corrosion behavior of each phase at different stages can be observed. After 5 cycles, the coating still maintained a relatively intact structure, with no obvious signs of corrosion. However, when the corrosion cycles extended to 100 and 120 cycles, selective corrosion occurred within the coating’s microstructure. Specifically, the Al-Zn eutectoid structure region in the coating showed obvious corrosion, while the α-Al phase, MgZn_2_ intermetallic compound, and ternary eutectic structure remained relatively stable at this stage, with no significant corrosion observed.

Further observing from Figure 11d, it can be seen that the Al-Zn eutectoid structure located on the shell of the aluminum dendrites not only preferentially corrodes and dissolves but also forms continuous corrosion channels within the coating. These channels provide pathways for the penetration and diffusion of air and H_2_O, thereby accelerating the corrosion process. This phenomenon further indicates that in the high-magnesium Zn-Al-Mg coating system, the Al-Zn eutectoid structure acts as a corrosion-sensitive phase, and its preferential corrosion behavior has an important influence on the failure mechanism of the coating under long-term cyclic corrosion environments.

## 4. Conclusions

Based on laboratory-prepared high-magnesium Zn-Al-Mg coated steel sheets with different Al contents, this paper systematically investigated the influence of the Al element on coating corrosion resistance and its mechanism. The main conclusions are as follows:

1. Cyclic corrosion test results show that the 19Al6Mg alloy coating exhibits the best planar corrosion resistance. Higher Al content in the coating does not mean better corrosion resistance. When the Al content in the coating reaches 22%, its planar corrosion resistance decreases.
2. Cut-edge corrosion test and punched Hole corrosion test results show that the 15Al6Mg alloy coating exhibits the best corrosion resistance. Under the same Mg content, as Al content increases, both the cut-edge corrosion resistance and punched Hole corrosion resistance of the coating show a decreasing trend.
3. The types of corrosion products formed on Zn-Al-Mg coatings with different Al contents are basically the same, but the cycles at which each corrosion product appears differ. As the Al content in the coating increases, the proportion of the Al phase and the resulting Al-Zn eutectoid structure in the microstructure increases. During the corrosion process, the Al-Zn eutectoid structure with lower corrosion potential corrodes preferentially, which is the main reason why increasing Al to 22% leads to a decrease in the overall corrosion resistance of the coating.

## Figures and Tables

**Table 1:** Chemical composition. Unit: wt%.

Elements	C	Si	Mn	P	S
/	0.04	0.020	0.20	0.010	0.008

**Table 2:** The melting points of different alloy components and bath temperature.

No.	Alloy Composition	Melting Point/°C	Bath Temperature/°C
1	Zn-15%Al-6%Mg	443	490
2	Zn-17%Al-6%Mg	452	495
3	Zn-19%Al-6%Mg	461	500
4	Zn-22%Al-6%Mg	472	515

**Table 3:** Hot-dip parameters for alloy-coated steel with different compositions.

Sample	Steel Temperature Before Zinc Bath/°C	Immersion Time/s	Cooling Rate After Zinc Bath/°C/s
15Al6Mg	510	3	10
17Al6Mg	515	3	10
19Al6Mg	520	3	10
22Al6Mg	535	3	10

**Table 4:** Corrosion potential of each phase in Zn-Al-Mg coating [19,34,35].

Phase	Concentration (Mass%)			V(S.C.E)
	Al	Zn	Mg	
Fe	-	-	-	−0.59
Al(Al_2_O_3_)	100	0	-	−0.78
55%Al-Zn	55	45	-	−1.00
Al-78%Zn	22	78	-	−1.1
Zn	0	100	-	−1.03
Al	100	0	-	−1.12
MgZn_2_	-	84	16	−1.23~−0.98

## Data Availability

The original contributions presented in this study are included in the article. Further inquiries can be directed to the corresponding author.
